# HIV As Trojan Exosome: Immunological Paradox Explained?

**DOI:** 10.3389/fimmu.2017.01715

**Published:** 2017-12-01

**Authors:** James E. K. Hildreth

**Affiliations:** ^1^Department of Internal Medicine, School of Medicine, Meharry Medical College, Nashville, TN, United States

**Keywords:** HIV, exosomes, antigen presentation, HIV vaccines, immune modulation

## Abstract

The HIV pandemic is still a major global challenge, despite the widespread availability of antiretroviral drugs. An effective vaccine would be the ideal approach to bringing the pandemic to an end. However, developing an effective HIV vaccine has proven to be an elusive goal. Three major human HIV vaccine trials revealed a strong trend toward greater risk of infection among vaccine recipients versus controls. A similar observation was made in a macaque SIV vaccine study. The mechanism explaining this phenomenon is not known. Here, a model is presented that may explain the troubling results of vaccine studies and an immunological paradox of HIV pathogenesis: preferential infection of HIV-specific T cells. The central hypothesis of this perspective is that as “Trojan exosomes” HIV particles can directly activate HIV-specific T cells enhancing their susceptibility to infection. Understanding the biology of HIV as an exosome may provide insights that enable novel approaches to vaccine development.

Carnathan et al. (1) employed the SIV/macaque model to evaluate immunization regimens under investigation in preclinical and clinical HIV vaccine studies. Their vaccine vectors did not include the envelope gene and could not induce neutralizing antibodies. Thus, the study specifically evaluated the role of cellular immune responses in protection against virus acquisition or control of replication after infection. All groups of animals showed SIV-specific CD8 T cells, but there was no correlation between the function or number of such cells and infection after rectal virus challenge. Notably, there were much higher levels of activated memory CD4 T cells in rectal biopsies from infected animals than from animals that remained negative after challenge. These data support the hypothesis that mucosal levels of activated CD4+ CCR+ T cells in virus-exposed animals predict the risk of virus acquisition.

It is well established that HIV preferentially infects activated memory CD4 T cells (2). For this reason, any factor that increases the number of such cells in mucosal tissues exposed to HIV may increase the risk of virus acquisition. Other sexually transmitted infections that cause accumulation of mucosal inflammatory cells are among such factors (3). Preferential infection of activated CD4 T cells may also explain sequential loss of CD4 T cells of defined specificities. For example, Loré et al. demonstrated that HIV-infected dendritic cells (DCs) can present CMV antigens and activate CMV-specific CD4 T cells. In their study, HIV was transmitted to activated CMV-specific T cells but not to non-responding T cells (4). These observations raise the possibility that any HIV vaccine that induces strong CD4 T cell responses may increase risk of transmission. As noted above, results from three major clinical vaccine trials evaluating multiple vaccine regimens—the HVTN-505, Phambili, and STEP trials—showed a strong trend toward greater risk of HIV acquisition among vaccine recipients versus placebo recipients (5–7). The mechanism explaining this troubling observation is unknown, but may be simply that the vaccines increased the pool of memory CD4 T cells in mucosal tissues. Interestingly, when Douek et al. examined the specificity of HIV-infected memory T cells from infected individuals, the data revealed that HIV preferentially infects HIV-specific CD4 T cells (8). These results suggest a peculiar immunological paradox for HIV: targeted infection of the very T cells that are programmed to respond to it. This phenomenon also predicts that vaccination regimens that increase the pool of mucosal HIV-specific CD4 T cells may result in greater risk of virus acquisition consistent with the trend toward higher risk of infection among HIV vaccine recipients (5–7).

One aspect of HIV biology may partly explain the paradoxical higher risk of HIV infection among vaccine recipients and preferential infection of HIV-specific T cells. Previously, we proposed that HIV is a Trojan exosomes (9). This model reconciled the complex release pathway of HIV, its somewhat unique lipid composition, and its host protein phenotype. Furthermore, the model predicted that HIV vaccines might result in higher risk of infection due to the virus’ ability to exploit cellular immune responses. Considerable data demonstrate the striking biochemical and biological parallels between HIV and exosomes (9–13). Exosomes are small virus-sized vesicles produced in late endosomes and released extracellularly by many cell types, including T cells, macrophages, and DC (11, 14). They package a variety of biologically relevant molecules and appear to function as a means of intercellular communication (15). Exosomes, like HIV, express a wide range of proteins on their surfaces. In both cases, the protein phenotype appears to reflect biogenesis from lipid rafts (15, 16) and includes adhesion molecules, major histocompatibility complex (MHC) proteins, and other proteins with immunological functions, including costimulatory molecules (15). In 2003, Hwang et al. showed that peptide-pulsed exosomes from DC could activate peptide-specific memory T cells in the absence of DC or added cytokines (17). A number of studies have confirmed that exosomes released from antigen-presenting cells are capable of activating memory T cells in the absence of the releasing cells (18, 19). Antigen-presenting cells, including macrophages, DC, and activated T cells, are major host cells for HIV.

Typically, after viruses infects cells or are endocytosed, viral proteins are processed and viral peptides are loaded onto MHC proteins for presentation to T cells (20). HIV acquires large numbers of both class I (HLA-A, B, and C) and class II (HLA-DR) MHC proteins from cells (21, 22) and some acquired HLA proteins are likely complexed with peptides from HIV proteins. This would allow HIV to present its own peptides to HIV-specific T cells and activate them. In the case of HIV peptide/class II MHC complexes, the activated CD4+ T cells could then be productively infected by the virus (Figure 1). Since the predominant fate of HIV-infected activated T cells is death, HIV would eliminate cells that are key to immune responses against it. HIV-infected cells are known to release exosomes (23) and they also could potentially activate HIV-specific T cells, making them susceptible to infection. This model would explain the high frequency of HIV-specific T cells among infected T cells *in vivo* (8). Also, virus-associated HLA class I molecules loaded with HIV peptides could activate HIV-specific CD8 T cells and contribute to chronic activation of these cells in untreated patients (24). It could also explain the higher risk of HIV acquisition in HIV-vaccinated individuals, especially if the vaccine results in HIV-specific memory CD4 T cells at sites of HIV exposure. All aspects of the proposed model are consistent with published data, with an important exception: the nature of HLA-associated peptides on HIV has not been investigated. Given the importance of developing an effective HIV vaccine and the troubling paradoxical infections observed in HIV vaccine studies, such studies are highly warranted. The results of such studies could provide deeper understanding of HIV immunopathogenesis and valuable insights regarding epitope selection for HIV vaccine candidates.

**Figure 1:**
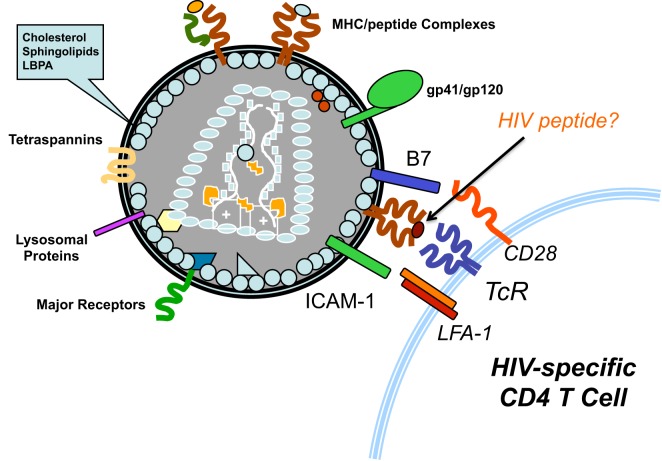
Activation of HIV-specific T cells by HIV. As Trojan exosome, HIV-1 acquires the proteins necessary to mediate antigen presentation to T cells. Emerging from HIV-infected antigen-presenting cells, HIV particles are likely to display class II HLA molecules loaded with HIV-derived peptides. Thus, the virus could activate HIV-specific CD4 T cells, making them highly susceptible to infection by the virus.

## Author Contributions

JH conceived the ideas and hypothesis and wrote the manuscript.

## Conflict of Interest Statement

The author declares that the research was conducted in the absence of any commercial or financial relationships that could be construed as a potential conflict of interest.
